# Rising in the World

**Published:** 1849-07

**Authors:** 


### RISING IN THE WORLD.

There is no such thing as *rising* except in honor and worth A man may commence life filing saws. He files his saws faithfully. He is a temperate, honest, and worthy man, as much so as is possible for him. By and by he finds he has a capacity to file other things than saws; and further on, by mending with files, he learns to make with files; still further he becomes a machinist, and making, learns to improve and invent. He takes out patents—becomes in his business the most noted, practical man of his time, and his friends to show their respect for him, send him to the Legislature, to Congress, and forthwith certain fools talk of his *rising* from a saw filer to a member of Congress! *Simply his sphere of usefulness was extended*, and just as much as he is less proud, more humble, more honest and faithful he is exalted, he has risen, and no more. If people knew more of what they call *rising*, they would give it another name. How many a man, who has *risen* to what the world calls the highest stations, looks back from the topmost round and sees that, so far as all the essentials of manhood are concerned, every step up has been two steps down; and who would give all his high honor, if it were possible for him to *rise* to his old station of a happy and honest saw filer.

It were better for us all to understand the matter of Profession and Station; to know and believe that the man dignifies the station, and not the station the man; that to him who honestly and faithfully performs his part, whatever it may be, consideration, respect and honor are due, and to teach the rising generation to trust to these last, and not to mere station, for credit and esteem.—[Lou. Ed.

---

How many minds—almost all the great ones—were formed in secresy and solitude, without knowing whether they should ever make a figure or not! All they knew was, they liked what they were about, and gave their whole souls to it.

